# Time-dependent effects of prone position on ventilation-perfusion matching assessed by electrical impedance tomography in patients with COVID-19 ARDS: sub-analysis of a prospective physiological study

**DOI:** 10.1186/s13613-025-01452-0

**Published:** 2025-03-31

**Authors:** Yuxian Wang, Yaxiaerjiang Muhetaer, Xin Zheng, Wei Wu, Jiale Tao, Ling Zhu, Jieqiong Song, Zhanqi Zhao, Ming Zhong

**Affiliations:** 1 Department of Critical Care Medicine, Zhongshan Hospital, Fudan University, Shanghai, 200032 China; 2 School of Biomedical Engineering, Guangzhou Medical University, Guangzhou, China; 3 Department of Critical Care Medicine, First Affiliated Hospital, Guangzhou Medical University, Guangzhou, China; 4 Department of Critical Care Medicine, Peking Union Medical College Hospital, Chinese Academy of Medical Sciences, Beijing, China; 5 Institute of Technical Medicine, Furtwangen University, Villingen-Schwenningen, Germany; 6 Shanghai Institute of Infectious Disease and Biosecurity, School of Public Health, Fudan University, Shanghai, China

**Keywords:** Electrical impedance tomography, Prone positioning, Acute respiratory distress syndrome, Ventilation-perfusion matching, Mechanical ventilation, Pulmonary perfusion

## Abstract

**Background:**

Prone positioning (PP) has been shown to improve oxygenation in patients with acute respiratory distress syndrome (ARDS); with a focus on its early physiological effects. However, the time-dependent effects of PP on ventilation-perfusion (V/Q) matching have not been fully investigated. In this study we aimed to investigate the longitudinal effects of PP on regional V/Q matching and the distribution of ventilation and perfusion in patients with coronavirus disease 2019 (COVID-19)-associated ARDS.

**Methods:**

This study analyzed patients with COVID-19 ARDS who were mechanically ventilated and underwent their first PP treatment. V/Q mismatching was assessed using electrical impedance tomography (EIT). At five intervals during the initial PP session PaO_2_/FiO_2_ measurements and EIT evaluations were performed including: before the initiation of PP while in the supine position (SP), 1 h after PP (PP_1_), 3 h after PP (PP_3_), 16 h after PP (PP_end_), and 3 h after reverting to the supine position (RE-SP_3_).

**Results:**

In this study eighteen COVID-19 ARDS patients were enrolled. In comparison with SP, PP led to significant improvements in oxygenation, with PaO_2_/FiO_2_ consistently increasing at each PP time point and peaking at PP_end_. Dorsal ventilation significantly increased at PP_1_ (*P* = .047), and steadily rose during PP, with a higher increase at PP_end_ than PP_1_ (*P* < .001). Dorsal perfusion remained unchanged during the first three hours of PP; however, significantly increased by PP_end_. Ventilation and perfusion returned to their baseline levels at RE-SP_3_. PP increased normal V/Q (%), and decreased non-perfused (%), low V/Q (%), particularly in the dorsal lung regions, compared with SP. At RE-SP_3_, there was a marked increase in the non-ventilated (%), low V/Q (%), and non-perfused (%) compared with PP. The global inhomogeneity (GI)-V/Q ratio was noted to have decreased during PP and correlated with an increase in PaO_2_/FiO_2_.

**Conclusions:**

In COVID-19-induced ARDS patients, prone positioning initially improves oxygenation and V/Q matching by enhancing ventilation distribution and decreasing low V/Q (%). Over time, perfusion changes further improve V/Q matching, but these benefits diminish once the patient returns to the supine position, leading to increased V/Q mismatch.

*Trial registration* Clinical Trials.gov, NCT04725227. Registered 25 January 2021, https://clinicaltrials.gov/study/NCT04725227?cond=NCT04725227&rank=1

**Supplementary Information:**

The online version contains supplementary material available at 10.1186/s13613-025-01452-0.

## Background

Prolonged prone positioning (PP) is an essential intervention that significantly reduces the mortality rate in moderate-to-severe acute respiratory distress syndrome (ARDS) [1–3]. Physiologically, PP improves oxygenation and mitigates ventilator-induced lung injury (VILI) by enhancing alveolar recruitment, reducing hyperinflation, and promoting a more uniform lung ventilation, thereby optimizing ventilation-perfusion matching [4–9]. However, despite the widespread recognition of these physiological findings, direct evidence of the short- and long-term effects of PP remains scarce in human studies.

Electrical impedance tomography (EIT) is a radiation-free, noninvasive technique enabling the imaging of regional impedance distribution in a cross-sectional area of the chest. EIT enables real-time visualization of regional lung ventilation and perfusion [10], making it an invaluable tool for assessing changes in V/Q matching during therapeutic interventions such as PP [9, 11–13]. Recent researches in EIT have introduced some methods to quantify pixel-level V/Q ratios, accounting for variables like cardiac output and minute ventilation [14–16]. Furthermore, Mauri et al. proposed a non-invasive correction that uses the pulsatility signal by EIT [16].

Previously, we demonstrated the benefits of PP in regional V/Q matching in patients with ARDS using EIT [9]. However, these findings were primarily derived from patients with ARDS without COVID-19. The pathophysiological characteristics of COVID-19-induced ARDS include high levels of dead space and distinct pulmonary vascular involvement. The study protocol and ethical approval were reviewed annually. The study is ongoing. Due to the pandemic, we screened COVID-19-associated ARDS patients and were able to include eligible subjects into the study. For the present study, we used a EIT- based analysis to quantify the pixels of different values of V/Q$\text{ ADDIN EN}.\text{CITE }$.

The current study aims to elucidate the longitudinal effects of PP on regional V/Q matching and the distribution of ventilation and perfusion in patients with COVID-19-associated ARDS. We hypothesize that PP induces time-dependent changes in both ventilation and blood flow, leading to improved V/Q matching and oxygenation. By leveraging EIT- based analysis, we seek to provide a detailed characterization of these physiological alterations throughout the duration of PP.

## Materials and methods

### Study design, setting, and participants

This prospective study was conducted in the intensive care unit (ICU) of Zhongshan Hospital, Fudan University, and approved by the Institutional Ethics Committee (NO. B2019-230(2)R). This study is an analysis of COVID-19 ARDS within an ongoing prospective clinical trial. Patients with suspected cases of COVID-19 were included after the diagnosis of COVID-19 was confirmed via reverse transcription polymerase chain reaction (RT-PCR) testing of nasal swabs. Written informed consent was obtained from the next-of-kin for all patients, in accordance with the ethical standards outlined in the Helsinki Declaration.

The inclusion criteria were: (1) patients under invasive mechanical ventilation; (2) moderate to severe ARDS as defined by the Berlin criteria [17]; (3) underwent the first PP session based on clinical judgment. The exclusion criteria included the following: younger than 18 years of age; classified as ARDS for more than 7 days before inclusion; had severe hemodynamic instability; were pregnant; underwent PP for less than 12 h; had contraindications to EIT (e.g., presence of a pacemaker or an inability to properly place the EIT belt due to chest surgical wounds dressing in the chest). This study was registered at ClinicalTrials.gov (NCT04725227).

### Study protocol

Upon enrollment, baseline characteristics were recorded, including height and weight, Acute Physiology and Chronic Health Evaluation II (APACHE II) score at ICU admission, ARDS etiology, and the ratio of arterial partial oxygen pressure to fractional concentration of inspired oxygen (PaO_2_/FiO_2_). Thoracic CT scan was performed in supine position (SP) before PP began.

EIT assessment and arterial blood gas (ABG) analyses were conducted at various time points during PP: before PP (SP), 1 h after PP (PP_1_), 3 h after PP (PP_3_), 16 h after PP (PP_end_), and 3 h after returning to supine position (RE-SP_3_). Respiratory and hemodynamic parameters, including heart rate (HR), central venous pressure (CVP), mean arterial pressure (MAP) and ventilator settings were recorded at each time point.

Under deep sedation and paralysis, mechanical ventilation was commenced using the Synchronized Intermittent Mandatory Ventilation (SIMV) mode. Ventilator settings were standardized across all patients including a tidal volume (Vt) ≤ 6 ml/kg predicted body weight, a constant inspiratory flow rate of 50 l/min, and a driving pressure maintained at ≤ 15cmH_2_O. The respiratory rate was adjusted at pH 7.35 to 7.45, and positive end-expiratory pressure (PEEP) was set after a recruitment maneuver (40cmH_2_O for 40 s) and decremental PEEP titration to achieve the highest compliance of respiratory system in the supine and prone position.

### EIT data collection and analysis

The EIT belt, fitted with 16 electrodes, was positioned around the chest wall at the fourth or fifth intercostal space and connected to an EIT monitor (PulmoVista 500; Dräger Medical GmbH, Lübeck, Germany). The belt remained in the same position throughout both supine and prone positioning. Technical details regarding the EIT have been previously documented [18]. EIT signals were captured at a frame rate of 50 Hz. Following the five-minute baseline EIT data recording, a 20-s end-inspiratory breath-hold was conducted. During this hold, 10 ml of 5% NaCl solution was rapidly injected via the central venous catheter. In less than two seconds injection was completed, resulting in a first-pass kinetic impedance dilution curve through the pulmonary circulation [14, 19].

1. The EIT data were analyzed using customized software with MATLAB R2023a (MathWorks, Natick, MA, USA) to qualify the following: Pixel-level ventilation: Measured as the impedance change between expiration and inspiration[20]. Pixels were classified as non-ventilated if the pixel ventilation was ≤ 10% of the highest pixel-level value measured in that patient.
2. Relative pixel-level perfusion: After preprocessing, the relative pixel perfusion, was obtained by normalizing each pixel’s steepest slope of the temporal EIT signal during saline bolus injection[20]. Pixels were classified as non-perfused if the pixel perfusion was ≤ 10% of the highest pixel-level value measured.
3. Percentage of ventilated or perfused pixels in region of interest (ROI): Pixels were across four ROIs (ventral to dorsal direction: ROI 1, ROI 2, ROI 3 and ROI 4) and the combined dorsal regions (ROI3 + ROI4) (Additional file 1: Figure S1).
4. Two types of V/Q matching were performed:
  The relative V/Q (V/Q-Rel) was obtained by dividing the percentage of ventilation by perfusion for each pixel [16].
  “Corrected” V/Q (V/Q-Corr): Adjusted V/Q-Rel values using EIT-based pulsatility data [16].
  The ventilation/perfusion matching was classified into five categories:
  Non-ventilated units (V/Q ratio ≤ 0.1);
  Low V/Q units (V/Q ratio 0.1–0.8);
  Normal V/Q units (V/Q ratio 0.8–1.25);
  High V/Q units (V/Q ratio 1.25–10);
  Non-perfused units (V/Q ratio ≥ 10).
5. $Wasted ventilation= \sum_{i=1}^{n}\left({\text{lg}\left(\frac{V}{Q}\right)}_{i}*{V}_{i}\right)\text{ ADDIN EN}.\text{CITE }$, where n represents the number of pixels in the functional EIT image within each ROI, considering units with a ​$\frac{V}{Q}$ ratio > 1.
6. $Wasted perfusion= \sum_{i=1}^{n}\left({-\text{lg}\left(\frac{V}{Q}\right)}_{i}*{Q}_{i}\right)$ [14], for the ​$\frac{V}{Q}$ ratio < 1.
7. Inhomogeneity of V/Q distribution is quantified using the global inhomogeneity (GI) index [21].

### Statistical analysis

The sample size was similar to previous physiologic studies [22, 23]. Statistical analyses were conducted using SPSS software (version 26.0; SPSS Inc. Chicago, IL, USA) and Prism 8 (GraphPad Software, San Diego, CA, USA). Shapiro–Wilk test was used to determine normality for all continuous variables. Data were presented as mean ± SD if normality was met, or otherwise as median and interquartile range. Repeated measures ANOVA with post-hoc Bonferroni correction was used to analyze the effect of time points on the variables. Mauchly’s test was conducted for sphericity, and the Greenhouse–Geisser correction was applied when sphericity was violated (p < 0.05). All statistical tests were two-tailed, and p < 0.05 was considered statistically significant.

## Results

### Patient characteristics

This study comprised eighteen COVID-19 ARDS patients who underwent mechanical ventilation in the ICU of Zhongshan Hospital, Fudan University, between December 2022 and March 2023. The primary features of the patients at inclusion are shown in Table 1. The cohort comprised 12 males and 6 females, with a mean age of 76.1 ± 11.2 years and an average body mass index (BMI) of 24.0 kg/m^2^. The mean APACHE II score at ICU admission was 16.1. All patients underwent continuous PP with an average duration of 16.78 ± 0.79 h. Baseline CT scans of patients are provided in the supplementary material (Additional file 1: Figure S2).

**Table 1:** Baseline characteristics of the patients

Patients’ characteristics (n = 18)	Mean ± SD or Median [25–75 percentiles] or n (%)
*Demographics*	
Age, years	76.1 ± 11.2
Male gender (%)	12 (66.7)
Body Mass Index, kg.m^−2^	24.0 ± 3.4
Comorbidities (%)	
Hypertension	11 (61)
Diabetes mellitus	6 (33)
Immunosuppression	3 (17)
*Disease severity*	
APACHE II score at ICU admission	16.1 ± 7.2
ARDS Etiology (%)	
COVID-19	18 (100)
ICU duration before enrollment, days	1 [0–2]
Intubation before enrollment, days	1 [1, 2]
Use of HFNC before intubation, n(%)	4 (22.2%)
Use of noninvasive ventilation before intubation, n(%)	3 (16.7%)
Duration of HFNC before intubation, d	3 ± 2
Duration of noninvasive MV before intubation, d	2 ± 1
*Clinical settings and gas exchange at enrollment*	
PEEP, cmH_2_O	7.6 ± 2.4
Tidal volume, ml.kg^−1^ PBW	6.0 ± 0.8
RR, min^−1^	20.9 ± 4.9
FiO_2_, %	66.4 ± 14.4
PaO2/FiO_2_, mmHg	127.2 ± 34.3
PaCO_2_, mmHg	50.1 ± 8.0
*Outcome*	
ICU length of stay	14.56 ± 9.78
Days of ventilation	13.61 ± 8.04
Survival	2Y/16N

APACHE-II, Acute Physiology and Chronic Health Evaluation II; ICU, intensive care unit; PaO_2_/FiO_2_, arterial partial pressure of O_2_/inspired fraction of O_2_ ratio; HFNC, high-flow nasal canula; PEEP, positive end-expiratory pressure; RR, Respiratory rate

### Improvement in oxygenation and hemodynamics

**Fig. 1:**
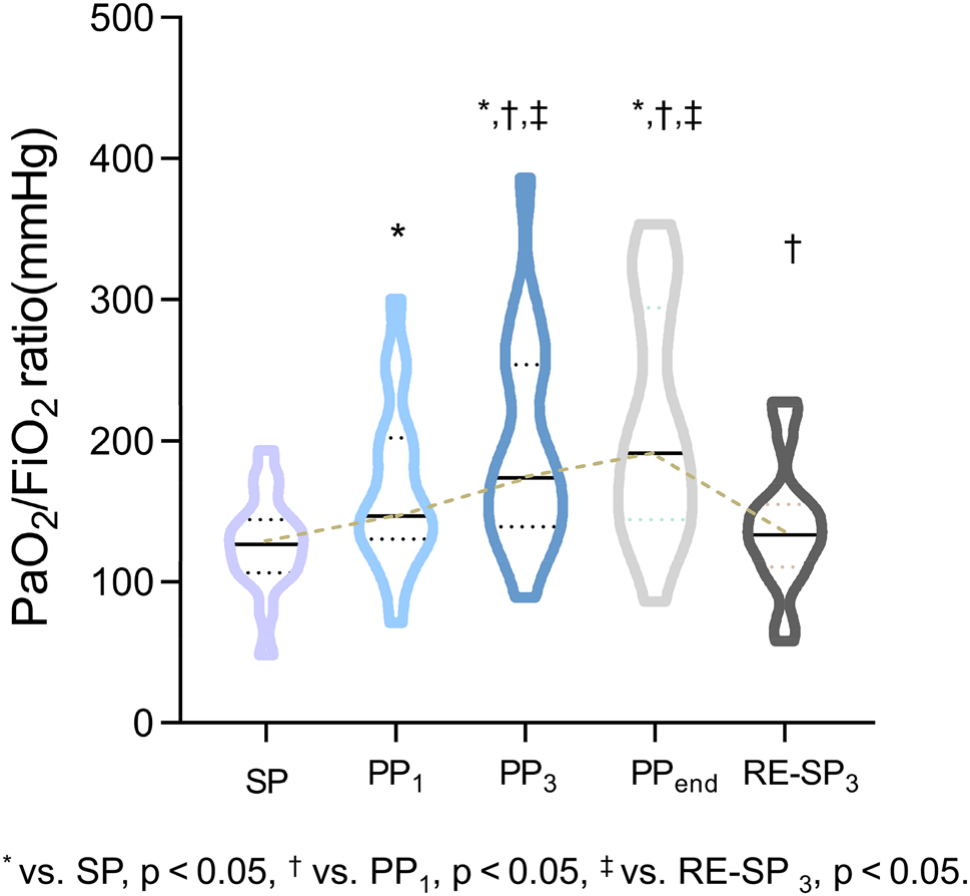
Evolution of PaO_2_/FiO_2_(mmHg) at before PP initiation (SP), 1 h after PP(PP_1_), 3 h after PP initiation (PP_3_), at the end of PP(PP_end_), and 3 h after returning to supine position (RE-SP_3_) within the same session. *vs. SP, *p* < 0.05, ^†^ vs. PP_1_, *p* < 0.05, ^‡^ vs. RE-SP_3_, *p* < 0.05

Clinical parameters regarding respiratory and hemodynamic status at SP, PP_1_, PP_3_, PP_end_ and RE-SP_3_ were evaluated and compared (Table 2). PP significantly enhanced oxygenation, demonstrated by the progressive increase in PaO_2_/FiO_2_ across the evaluated time points (127.22 vs. 166.22 vs. 196.74 vs. 211.39 vs. 139.00 mmHg, *p* < 0.001) (Table 2, Fig. 1). Although decreases in HR, SBP, MAP and norepinephrine doses were observed, these changes were not statistically significant (Table 2).

**Table 2:** Hemodynamics and blood gas analysis variables

Variables	SP	PP_1_	PP_3_	PP_end_	RE-SP_3_	Trend*-P* value
*Ventilator setting*						
FiO_2_, %	66.39 ± 14.43	64.17 ± 12.40	60.56 ± 10.42	60.83 ± 8.62	61.39 ± 15.03	0.016
VT, kg/ml	6.04 ± 1.37	5.89 ± 1.34	5.94 ± 1.34	5.90 ± 1.35	5.98 ± 1.38	0.184
RR	20.94 ± 5.38	22.33 ± 4.52	23.17 ± 4.38	22.94 ± 4.44	23.67 ± 4.50	0.008
PEEP, cmH_2_O	7.89 ± 2.56	7.28 ± 2.63	7.33 ± 2.57	7.00 ± 2.50	7.06 ± 2.26	0.093
*Respiratory mechanics*						
Plateau pressure, cmH_2_O	22.50 ± 4.01	22.61 ± 2.77	23.72 ± 3.72	23.00 ± 4.12	21.72 ± 3.48	0.268
Driving pressure, cmH_2_O	14.61 ± 3.35	15.33 ± 2.20	16.39 ± 3.48	16.00 ± 3.71	14.67 ± 3.82	0.268
Respiratory system quasi-static compliance, ml/cmH_2_O	28.87 ± 10.31	28.35 ± 11.97	25.21 ± 8.38	25.91 ± 9.56	29.28 ± 10.00	0.076
*Arterial blood gases*						
PaO_2_ (mmHg)	80.89 ± 16.41	102.48 ± 29.30^*^	113.49 ± 28.96^*,‡^	124.48 ± 41.10^*,†,‡^	79.93 ± 13.55†	< 0.001
PaO_2_/FiO_2_ (mmHg)	127.22 ± 34.29	166.22 ± 58.66^*^	196.74 ± 75.72^*,†,‡^	211.39 ± 82.06^*,†,‡^	139.00 ± 45.12^†^	< 0.001
PaCO_2_ (mmHg)	50.19 ± 8.03	50.83 ± 7.84	49.41 ± 6.79	49.55 ± 7.57	51.01 ± 7.90	0.796
*Hemodynamic variables*						
HR (bpm)	84.28 ± 19.96	84.44 ± 21.57	79.06 ± 18.09	78.33 ± 19.78	80.94 ± 21.38	0.529
SBP (mmHg)	132.83 ± 21.52	127.33 ± 20.74	123.94 ± 20.33	128.11 ± 24.16	125.22 ± 20.81	0.583
MAP (mmHg)	86.72 ± 11.31	85.56 ± 11.33	82.91 ± 12.35	83.30 ± 12.63	81.33 ± 12.22	0.404
Norepinephrine (µg/kg/min)	0.045[0–0.106]	0.024[0–0.127]	0[0–0.074]	0.015[0–0.106]	0.015[0–0.070]	0.755

PEEP, positive end-expiratory pressure; PaO_2_/FiO_2_, arterial partial pressure of O_2_/inspired fraction of O_2_ ratio; PaCO_2_, arterial partial pressure of CO_2_; HR, heart rate; SBP, systolic arterial blood pressure; MAP, mean arterial pressure. SP: in the supine position; PP_1_: 1 h after PP initiation; PP_3_: 3 h after PP initiation; PP_end_: at the end of PP; RE-SP_3_:3 h after supine position

p value by one-way analysis of variance (ANOVA) for repeated-measures

*vs. SP, *p* < 0.05, ^†^ vs. PP_1_, *p* < 0.05, ^‡^ vs. RE-SP_3_, *p* < 0.05

### Regional distribution of lung ventilation and perfusion

**Fig. 2:**
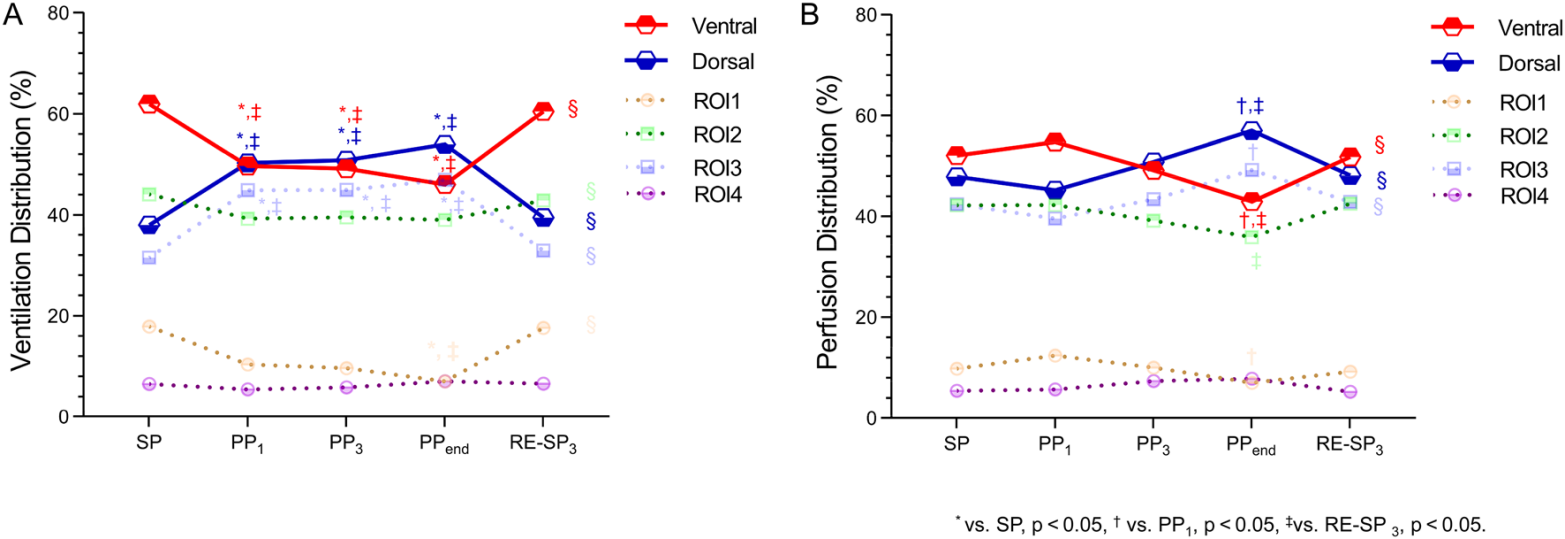
Evolution of ventral, dorsal, ROI 1-4 of ventilation (**A**) or perfusion distribution (%) (**B**) before PP initiation (SP), 1 h after PP (PP_1_), 3 h after PP initiation (PP_3_), at the end of PP (PP_end_), and 3 h after returning to supine position (RE-SP_3_) within the same session. *vs. SP, *p* < 0.05, ^†^ vs. PP_1_, *p* < 0.05, ^‡^ vs. RE-SP_3_, *p* < 0.05

The EIT-based measurements for lung ventilation and perfusion at the indicated time points are presented in Table 3 and Fig. 2A. Notably, dorsal ventilation significantly increased during PP (38.00 vs. 50.32 vs. 50.80 vs. 53.94 vs. 39.47%, *p* < 0.01). The increments primarily occurred in ROI 3, while no significant increment in ROI 4.

**Table 3:** EIT data variables

Variables	SP	PP_1_	PP_3_	PP_end_	RE-SP_3_	Trend*-P* value
*Ventilation*						
TIV	1940.00[1594.25–2224.50]	1980.50[1497.50–2533.75]	2293.50[1717.25–2526.75]	1907.50[1621.75–2442.00]	2132.00[1626.50–2530.00]	0.402
EELI	615.50[431.25–822.75]	503.50[392.75–686.00]	690.00[402.25–736.25]	492.00[396.00–764.75]	637.50[518.75–880.50]	0.593
Dorsal ventilation, %	38.00 ± 13.57	50.32 ± 9.62^*,‡^	50.80 ± 9.84^*,‡^	53.94 ± 12.11^*,‡^	39.48 ± 13.50	< 0.001
ROI 1 of ventilation distribution, %	17.89 ± 10.48	10.36 ± 7.26	9.65 ± 7.25	7.06 ± 4.97^*,‡^	17.62 ± 11.91	< 0.001
ROI 2 of ventilation distribution, %	44.11 ± 7.64	39.32 ± 5.29	39.55 ± 7.30	39.00 ± 9.60	42.91 ± 7.74	0.045
ROI 3 of ventilation distribution, %	31.53 ± 13.70	44.87 ± 8.24^*,‡^	44.94 ± 8.39^*,‡^	46.94 ± 9.35^*,‡^	32.91 ± 13.69	< 0.001
ROI 4 of ventilation distribution, %	6.47 ± 3.42	5.46 ± 3.71	5.86 ± 3.85	7.00 ± 4.66	6.57 ± 3.58	0.594
*Perfusion*						
Dorsal perfusion, %	47.89 ± 14.56	45.26 ± 13.45	50.81 ± 12.10	57.06 ± 12.06^†,‡^	48.18 ± 10.57	0.031
ROI 1 of perfusion distribution, %	9.85 ± 8.05	12.41 ± 8.75	10.03 ± 6.73	7.04 ± 4.70^†^	9.22 ± 3.87	0.074
ROI 2 of perfusion distribution, %	42.26 ± 7.57	42.33 ± 10.01	39.17 ± 8.84	35.89 ± 8.69^‡^	42.59 ± 8.23	0.101
ROI 3 of perfusion distribution, %	42.44 ± 12.64	39.57 ± 10.92	43.45 ± 9.43	49.26 ± 9.44^†^	42.93 ± 8.26	0.020
ROI 4 of perfusion distribution, %	5.45 ± 3.14	5.67 ± 3.01	7.36 ± 3.95	7.81 ± 3.84	5.25 ± 3.83	0.093
*Ventilation-perfusion matching- Relative*						
Pixels with						
Non-ventilated (V/Q ≤ 0.1), %	9.98 ± 7.01	11.38 ± 6.72	10.95 ± 7.63^‡^	9.38 ± 5.42^‡^	13.54 ± 6.75	0.375
Low V/Q (0.1 < V/Q < 0.8), %	25.09 ± 6.84	18.81 ± 6.32^*^	18.39 ± 8.87	16.55 ± 7.28^*,‡^	23.73 ± 5.97	< 0.001
Normal V/Q (0.8 ≤ V/Q ≤ 1.25), %	18.51 ± 7.85	25.28 ± 10.65	27.91 ± 11.88^*^	39.57 ± 12.99^*^^,^^†^^,^^‡^	20.41 ± 9.35	< 0.001
High V/Q (1.25 < V/Q < 10), %	14.90 ± 10.98	32.95 ± 14.07^*^	30.40 ± 13.99^*^	20.94 ± 13.29^†^	20.80 ± 13.60	0.002
Non-perfused (V/Q ≥ 10), %	31.52 ± 10.40	11.58 ± 9.72^*,‡^	12.27 ± 8.24^*,‡^	13.56 ± 9.14^*,‡^	21.52 ± 9.87^*,†^	< 0.001
Wasted ventilation, %	27.83 ± 12.16	17.50 ± 9.41	19.56 ± 12.95	17.78 ± 7.72^*,‡^	28.17 ± 15.39	0.008
Wasted perfusion, %	36.83 ± 11.49	26.72 ± 7.27^*^	23.67 ± 7.35^*,‡^	18.17 ± 7.20^*,†,‡^	34.50 ± 9.70	< 0.0001
GI V/Q	0.808 ± 0.089	0.696 ± 0.134^*^	0.695 ± 0.168	0.684 ± 0.134^*^	0.780 ± 0.104	< 0.001

TIV, Tidal Impedance Variation; EELI, End-Expiratory Lung Impedance; ROI, Region of Interest; GI, Global Inhomogeneity. SP: in the supine position; PP_1_: 1 h after PP initiation; PP_3_: 3 h after PP initiation; PP_end_: at the end of PP; RE-SP_3_:3 h after supine position

p value by one-way analysis of variance (ANOVA) for repeated-measures

*vs. SP, *p* < 0.05, ^†^ vs. PP_1_, *p* < 0.05, ^‡^vs. RE-SP_3_, *p* < 0.05

Perfusion in the dorsal region significantly increased with PP duration (47.89 vs. 45.26 vs. 50.81 vs. 57.06 vs. 48.18%, *p* < 0.05) (Table 3, Fig. 2B). Dorsal perfusion significantly increased at PP_end_ compared with PP_1_ (57.06 ± 12.06 vs. 42.26 ± 13.45%, *p* < 0.05), with a corresponding increase in ROI 3 (49.26 ± 9.44 vs, 39.57 ± 10.92%, *p* < 0.05) and a decrease in ROI 1 perfusion (7.04 ± 4.70 vs. 12.41 ± 8.75, *p* < 0.05). Following the return to the supine position, dorsal perfusion significantly decreased compared to PP_end_ (48.18 ± 10.57 vs. 57.06 ± 12.06, *p* < 0.05); however, ROI 2 perfusion increased (42.59 ± 8.23 vs. 35.89 ± 8.69, *p* < 0.05).

### Ventilation-perfusion matching

**Fig. 3:**
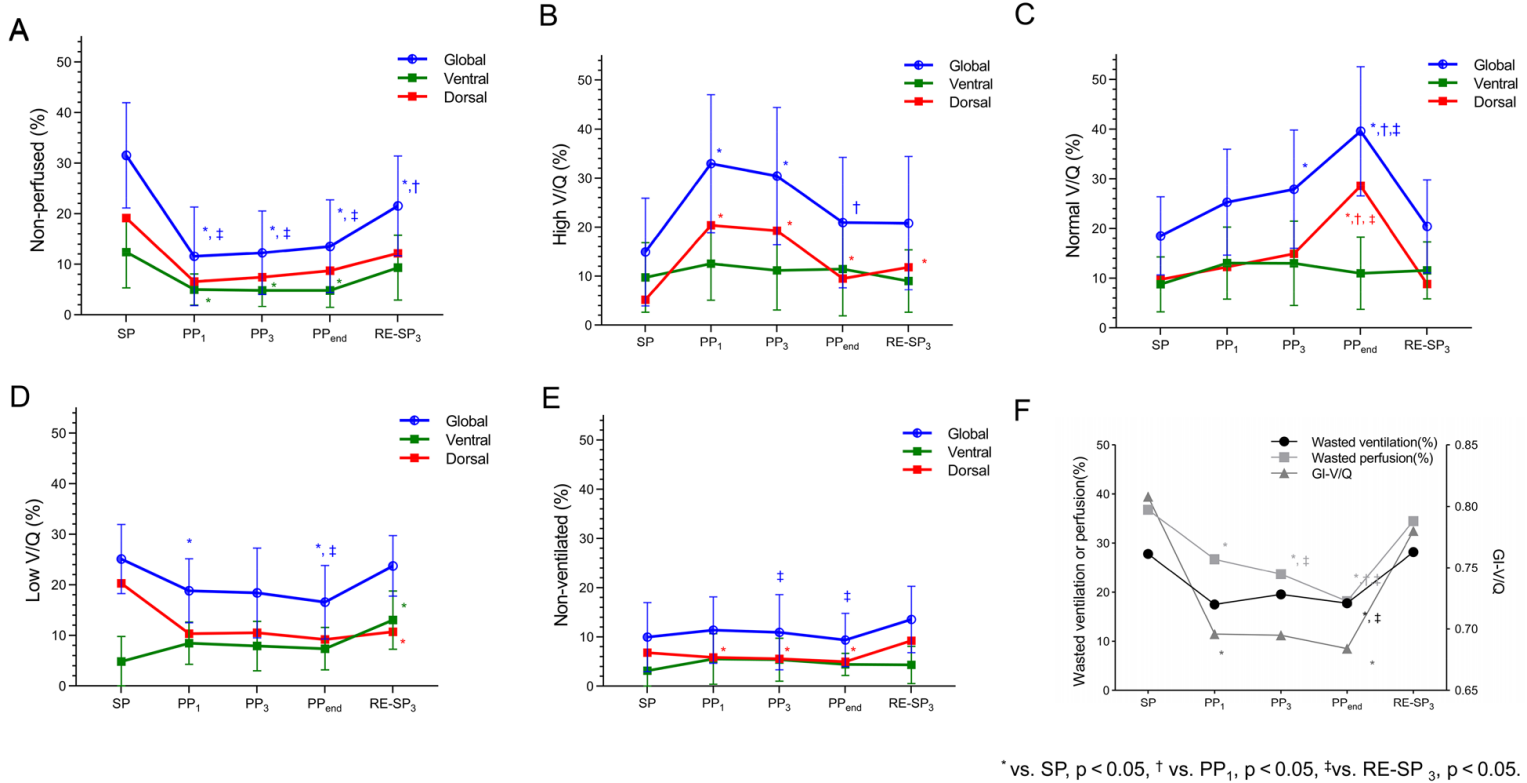
Comparisons of global, ventral and dorsal non-perfused (%) (**A**), high V/Q (%) (**B**), normal V/Q (%) (**C**), low V/Q (%) (**D**), and non-ventilated (%) (**E**) before PP initiation (SP), 1 h after PP (PP_1_), 3 h after PP initiation (PP_3_), at the end of PP (PP_end_), and 3 h after returning to supine position (RE-SP_3_) within the same session. Evolution of wasted ventilation (%), wasted perfusion (%) and GI-V/Q at SP, PP_1_, PP_3_, PP_end_, RE-SP_3_ (**F**). The V/Q ratio was determined by dividing the pixel-level ventilation obtained from electrical impedance tomography by the measured perfusion. *vs. SP, *p* < 0.05, ^†^vs. PP_1_, *p* < 0.05, ^‡^ vs. RE-SP_3_, *p* < 0.05

**Fig. 4:**
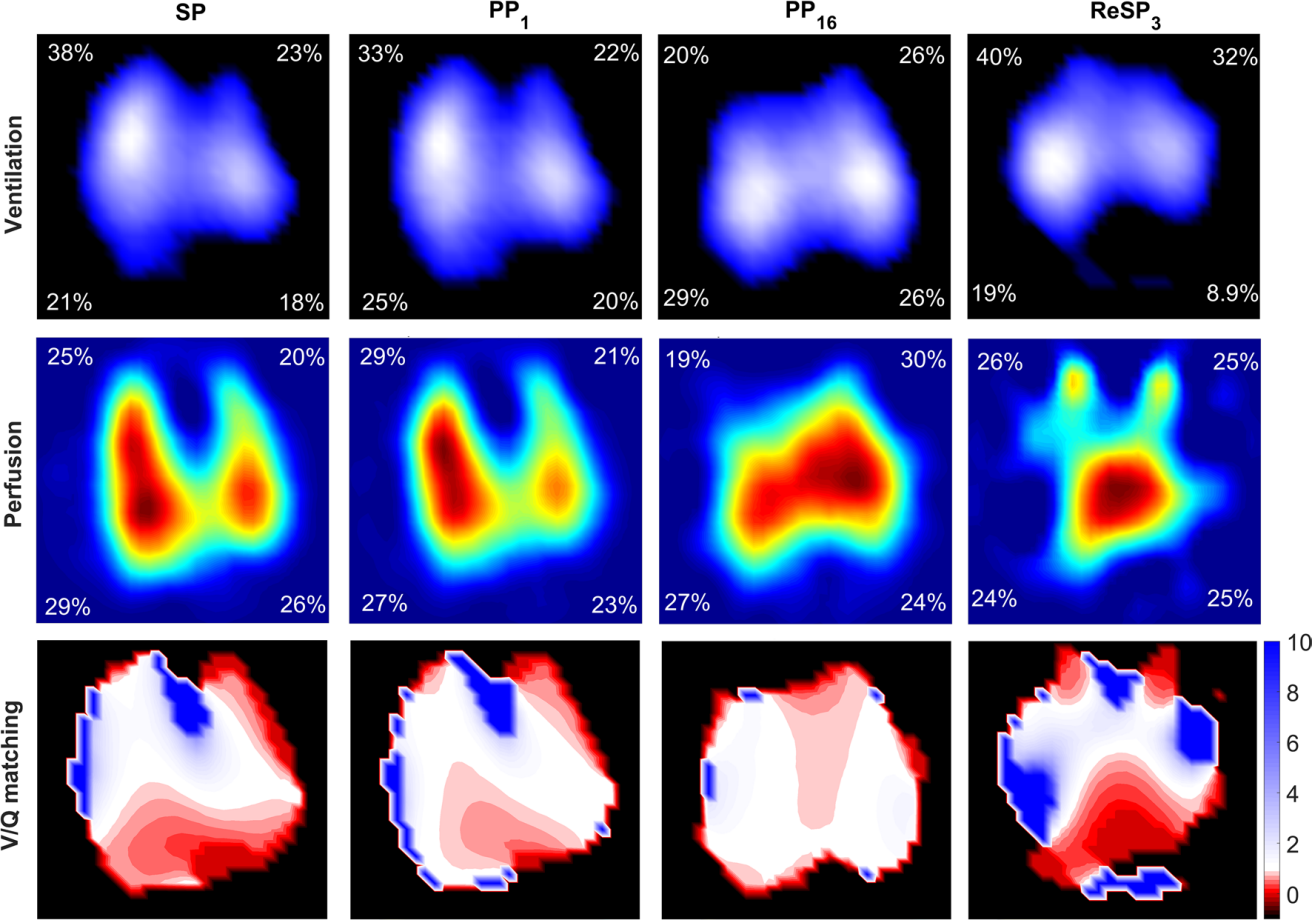
Effects of prone position on ventilation-perfusion matching in a representative study patient. Top map: image of the ventilation distribution. Middle map: image of perfusion distribution. Bottom map: obtained by integrating ventilation and perfusion maps-V/Q matching. The V/Q ranges from < 0.1 (non-ventilated units, indicated in red) to 1 (normal units, depicted in white) to > 10 (non-perfused units, represented in blue). The color scale is presented on the right side of the V/Q maps. SP: in the supine position; PP_1_: 1 h after PP initiation; PP_3_: 3 h after PP initiation; PP_end_: at the end of PP; RE-SP_3_:3 h after supine position

EIT measurements revealed significant improvements in V/Q matching during PP. Notably, non-perfused (%) decreased significantly throughout the PP session (31.52 vs. 11.58 vs. 12.27 vs. 13.78 vs. 21.52, *p* < 0.001) (Fig. 3A). High V/Q (%) increased at PP_1_ and decreased thereafter (14.90 vs. 32.95 vs. 30.40 vs. 20.94 vs. 20.80, *p* = 0.002) (Fig. 3B). Normal V/Q (%) significantly increased during PP (18.51 vs. 25.28 vs. 27.91 vs. 39.57 vs. 20.41, *p* < 0.001) (Fig. 3C). Non-ventilated (%) remained almost unchanged throughout PP (*p* = 0.375, Table 3, Fig. 3E), while low V/Q (%) significantly decreased (25.09 vs. 18.81 vs. 18.39 vs. 16.55 vs. 23.73, *p* < 0.001) (Fig. 3D). After a 3-h period of returning to the supine position, non-ventilated (%), low V/Q (%) and non-perfused (%) showed a significant increase compared with the PP (Fig. 3). Additional file 1: Figure S3 shows the regional distribution of ventilation and perfusion in the ventral and dorsal regions with different ranges of V/Q at SP, PP_end_, and RE-SP_3_. Regional changes in different V/Q values are summarized in the Additional file 1: Table S1-2 and Figure S4. Figure 4 shows the distribution of V/Q matching in a representative patient. This reveals a higher proportion of normal V/Q (depicted as white) and a lower number of non-perfused (blue) and non-ventilated (red) units throughout the pulmonary pathway.

During PP, wasted ventilation (%) and perfusion (%) significantly decreased particularly in the dorsal region (Additional file 1: Table S3). Moreover, the GI index of V/Q matching showed a significant decrease during the PP (*p* < 0.001) (Table 3, Fig. 3F). Similar trends were observed in the V/Q-Corr maps (Additional file 1: Table S3).

### Correlation analysis

**Fig. 5:**
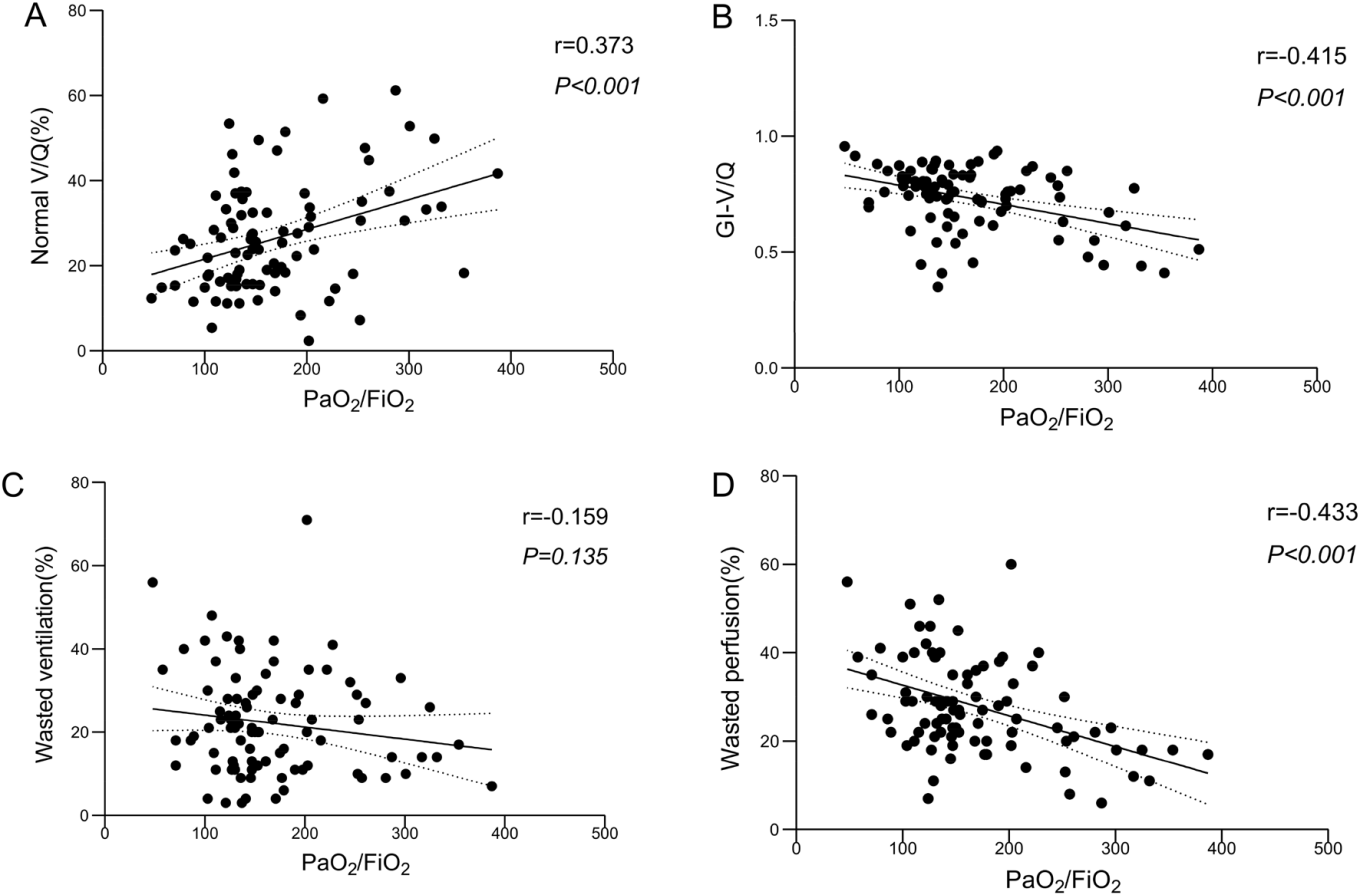
Correlation between PaO_2_/FiO_2_ and normal V/Q (%) (**A**), GI-V/Q (%) (**B**), Wasted ventilation (%) (**C**) and Wasted perfusion (%) (**D**). The V/Q ratio was determined by dividing the pixel-level ventilation obtained from electrical impedance tomography by the measured perfusion

PaO_2_/FiO_2_ exhibited a positive correlation with normal V/Q% (rho = 0.373, *p* < 0.001) and a negative correlation with both the GI-V/Q and wasted perfusion% (rho = − 0.415, *p* < 0.001; rho = − 0.433, *p* < 0.001) (Fig. 5). No correlation was found between PaO_2_/FiO_2_ and wasted ventilation (rho = − 0.159, *p* = 0.135) (Fig. 5C).

## Discussion

This study provides a comprehensive analysis of the time-dependent physiological effects of PP on ventilation-perfusion matching in COVID-19-induced ARDS using EIT. The key findings included the following: (1) PaO_2_/FiO_2_ exhibited immediate and rapid improvement at PP_1_ and PP_3_, and continued to increase at PP_end_. (2) Dorsal ventilation increased gradually at PP_1_, but a significant increase at PP_3_ and PP_end_. Dorsal blood flow began to improve at PP_3_ and this increase was sustained at PP_end_. (3) From PP_1_, the normal V/Q (%) began to increase and the non-perfused (%) began to decrease. This was sustained throughout the PP session. The high V/Q (%) increased at PP_1_ and gradually decreased. In the prone position, the non-ventilated (%) showed no significant changes, whereas there was a significant decrease in the low V/Q (%). (4) After reverting to the supine position for 3 h, the ventilation and perfusion distribution tended towards the SP state. There was a significant increase in non-ventilated (%), low V/Q (%), and non-perfused (%) compared to PP. (5) During PP, wasted ventilation (%) and perfusion (%) decreased significantly. Moreover, the GI index of V/Q matching showed a significant decrease during PP.

It is widely acknowledged that PP can directly influence gas distribution due to alterations in gravity, whereas blood flow distribution is primarily indicated by the anatomical structure and regional pulmonary vascular resistance, which are largely unaffected by gravity [5, 24]. Previous studies have shown that prolonged PP promotes the re-expansion of collapsed lung regions and improves ventilation, subsequently enhancing pulmonary perfusion, and optimizing ventilation-perfusion matching [9]. COVID-19 is a unique subtype of ARDS with significantly higher dead space [13, 19]; however, we discovered that PP improves PaO_2_/FiO_2_ in these patients, similar to non-COVID-19 ARDS patients. In this study, we observed that the improvement of dorsal ventilation was rapid, while blood flow gradually shifted towards the dorsal side during PP. Furthermore, our findings indicated that upon reverting to the supine position, ventilation and blood flow returned to baseline levels within three hours.

EIT measurements may provide imprecise estimates of V/Q compartments; however, longitudinal evaluation of regional V/Q matching is clinically valuable for tracking disease progression and assessing therapeutic interventions. The main strength of EIT-based V/Q lies in its ability to detect trends over time [25]. In this study, the proportion of normal V/Q (%) in the dorsal region significantly increased during prone positioning, while the proportion of normal V/Q (%) in the ventral region was maintained without any significant decrease. PP led to a significant reduction in non-perfused (%) in COVID-19 ARDS, including a notable decrease observed in the ventral non-perfused (%). Repositioning from PP to the supine position resulted in a slight decrease in non-perfused (%) compared with the baseline, while the normal V/Q (%) returned to a similar baseline level. Interestingly, the high V/Q (%) at PP_1_ initially increased and then trend to decrease. During the prone position, there was a significant decrease in the dorsal non-ventilated (%) and low V/Q (%). Despite no statistically significant difference in global non-ventilated (%), oxygenation steadily improved during the prone position, which may be attributed to the lower low V/Q (%) and wasted perfusion (%) [26]. After three hours in the re-supine position, the V/Q mismatch compared with the PP significantly increased. The aforementioned findings indicate that to obtain sustained improvement in gas exchange and V/Q matching, extended PP or multiple sessions of PP may require further studies [1, 27, 28].

Wasted ventilation (%) and perfusion (%) decreased in the PP, demonstrating that the PP significantly enhanced the exchange of gas and blood. Moreover, GI-V/Q was lower in the PP than in the supine position. In the prone position, the ventilation-perfusion ratio exhibits greater homogeneity. Reactivity to PP in ARDS patients with COVID-19 exhibits individual variations, potentially attributed to the temporal epidemic of different strains of COVID-19 or influenced by the distinct stages of ARDS. Perier et al. reported that after three hours of PP, lung aeration improved, and dorsal pulmonary perfusion remained unmodified in COVID-19-associated ARDS [11]. In contrast, it was found that after the transition from SP to PP, both ventilation and perfusion changed within one hour in a patient with COVID-19 ARDS [29]. Another clinical study reported that pronation for 30 min decreased the fraction of ventilated nonperfused units and reduced dead space/shunt ratio in the ventral lung regions of patients with COVID-19-associated ARDS [12]. Xin et al. reported the effects of PP on lung aeration may influenced by the stage of lung injury and the duration of prior ventilation, indicating the futility of PP if applied late [30].Furthermore, Rossi et al. revealed that patients with COVID-19 pneumonia who underwent assessments three weeks after admission had higher amounts of consolidated tissue, which determined the oxygenation responses following pronation and recruitment maneuvers [31]. Moreover, Yuan et al. discovered that PP significantly reduced V/Q mismatch in patients with early-stage ARDS, and increased the V/Q mismatch in patients with persistent (> 7 days) ARDS [32], of which more than 45% had COVID-19.

COVID-19-induced ARDS is a complex disorder characterized by various pathological features including tracheobronchitis, diffuse alveolar damage, and vascular injury. Pulmonary vessels exhibit inflammation, vasculitis, microthrombi, and occasionally macrothrombi. In contrast, viral infections in areas of ongoing active injury contribute to persistent and temporally heterogeneous lung damage. These virus-related pathophysiological injuries affect both alveoli and pulmonary vasculature to varying degrees, resulting in distinct phenotypes [33, 34]. This heterogeneity highlights the need for caution among ICU physicians when planning COVID-19 treatments solely on simplistic phenotypic models, and underscores the importance of adopting a more comprehensive and individualized approach to ventilatory management. In critically ill individuals, it is important to assess regional alterations in ventilation and perfusion using EIT.

This study has provided notable evidence regarding the short and long-term physiological effects of prone ventilation in patients with COVID-19-induced ARDS. However, it is important to acknowledge the study’s limitations. First, as an exploratory study with a limited sample size, our findings warrant confirmation. Second, EIT measures only a portion of the lung, giving a 2-dimensional image, which may not be sufficient for assessing the entire spectrum of ventilation-perfusion matching in the lung [35]. Third, due to technical and logistic challenges, particularly the increase in COVID-19 cases, cardiac output (CO) measurements were not obtained. As a result, the V/Q values measured by EIT were relative or corrected, not absolute. Additionally, we lack the calculation of dead space fraction and shunt fraction, which are also valuable indicators. Fourth, partitioned respiratory mechanics especially chest wall and lung compliances were not measured using esophageal pressure. This leaves the effects of the chest wall properties on PP unclear.

## Conclusions

In COVID-19-induced ARDS patients, prone positioning initially improves oxygenation and global V/Q matching by altering ventilation distribution, with these effects persisting throughout the session. Perfusion changes occur later in prone positioning, further enhancing oxygenation, particularly in the dorsal lung regions. V/Q distribution is more homogeneous in the prone position, but V/Q mismatching increases significantly after returning to the supine position. Further research is needed to explore the impact of extended or repeated prone positioning sessions.

## Supplementary Information

Supplementary material 1.

## Data Availability

The datasets used and/or analyzed during the current study are available from the corresponding author on reasonable request.

## Abbreviations

COVID-19: Coronavirus disease
ARDS: Acute respiratory distress syndrome
V/Q: Ventilation/perfusion
EIT: Electrical impedance tomography
SP: Supine position
PP: Prone position
RE-SP: Reverting to the supine position
VILI: Ventilator induced lung injury
RT-PCR: Reverse transcription polymerase chain reaction
PaO_2_: Arterial partial oxygen pressure
FiO_2_: Fractional concentration of inspired oxygen
SIMV: Synchronized intermittent mandatory ventilation
PEEP: Positive end-expiratory pressure
Vt: Tidal volume
RR: Respiratory rate
APACHE II: Acute physiology and chronic health evaluation II
ROI: Region of interest
