# Supplementary material for: Time-dependent effects of prone position on ventilation-perfusion matching assessed by electrical impedance tomography in patients with COVID-19 ARDS: sub-analysis of a prospective physiological study
Source: Ann Intensive Care. 2025 Mar 31;15:46. doi: 10.1186/s13613-025-01452-0 (PMC11958859; doi:10.1186/s13613-025-01452-0)
Supplement: Supplementary file 1 — Supplementary material 1. [file 13613_2025_1452_MOESM1_ESM.docx]

**Time-dependent effects of prone position** **on ventilation-perfusion matching assessed by electrical impedance tomography in patients with COVID-19 ARDS: a prospective physiological study**

Yu-xian Wang^#^^1^, BS; Yaxiaerjiang Muhetaer^#1^, BS; Xin Zheng^#1^, BS; Wei Wu^1^, MS; Jia-le Tao^1^, BS; Ling Zhu^1^, BS; Jie-qiong Song, PhD, MD; Zhanqi Zhao^*2.3.4^, PhD; Ming Zhong^*1,5,6^, PhD, MD

**Online supplement**

**Supplemental Figures**


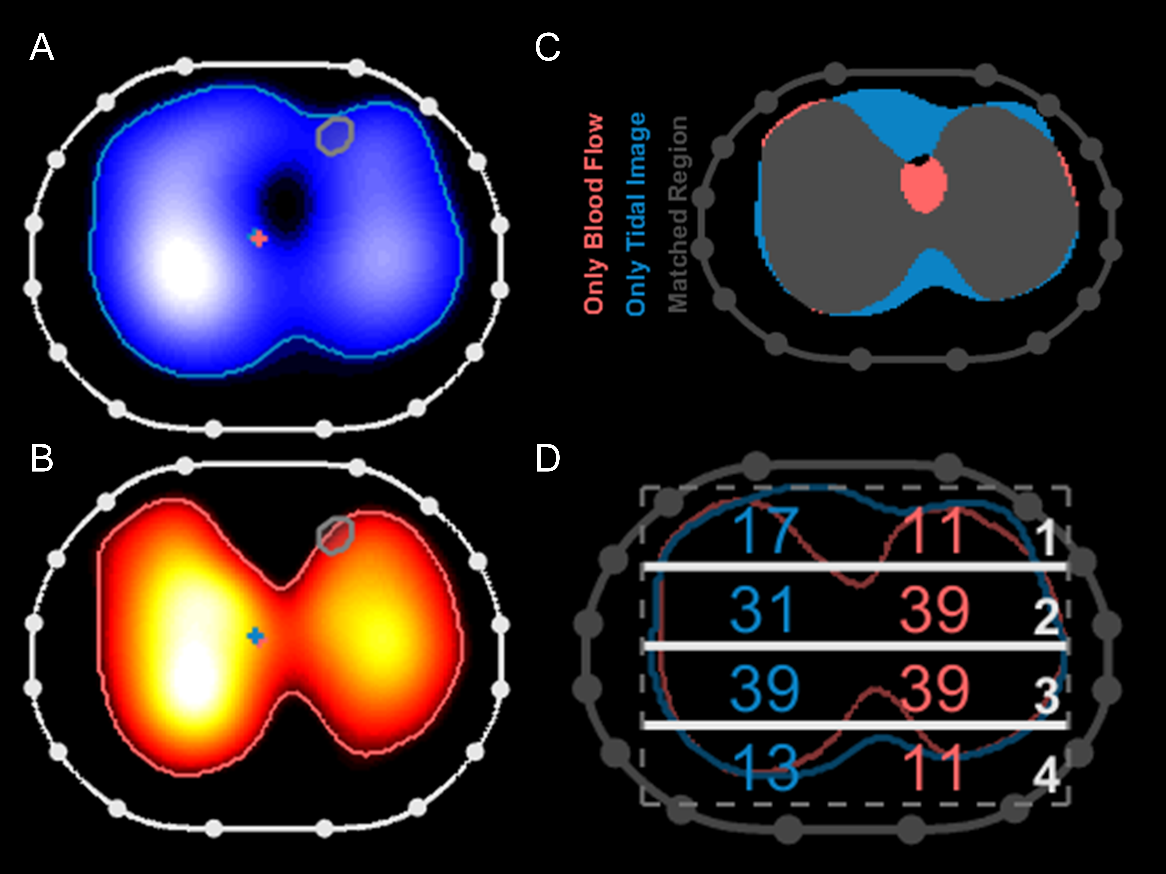


**Figure S1. Ventilation and perfusion measured by EIT in a representative patient.** A. Representative image of the ventilation (blue-color map) distribution. B. Representative image of the perfusion (red-color map) distribution. C. Representative map obtained by integrating ventilation and perfusion maps: the grey area indicates matched units which are both ventilated and perfused, while red area indicated only perfused units and blue area only ventilated units. D. Representative map with the percentage of ventilation (blue numbers) and perfusion (red numbers) distribution in the four horizontal regions of interest (ROIs). This choice allowed us to obtain more superimposable regions of interest.


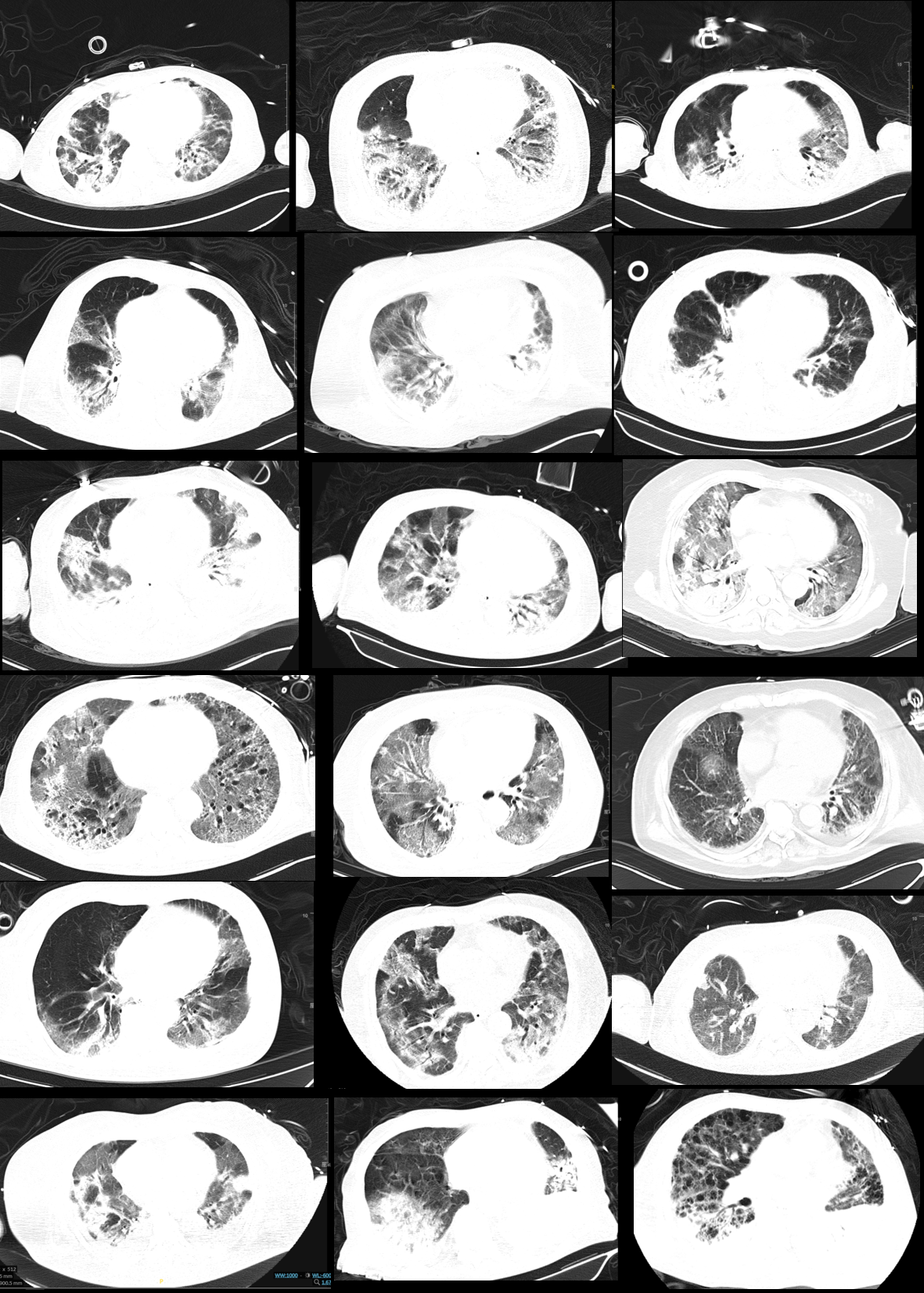
**Figure S2. CT scan images of eighteen patients before enrollment.**

**
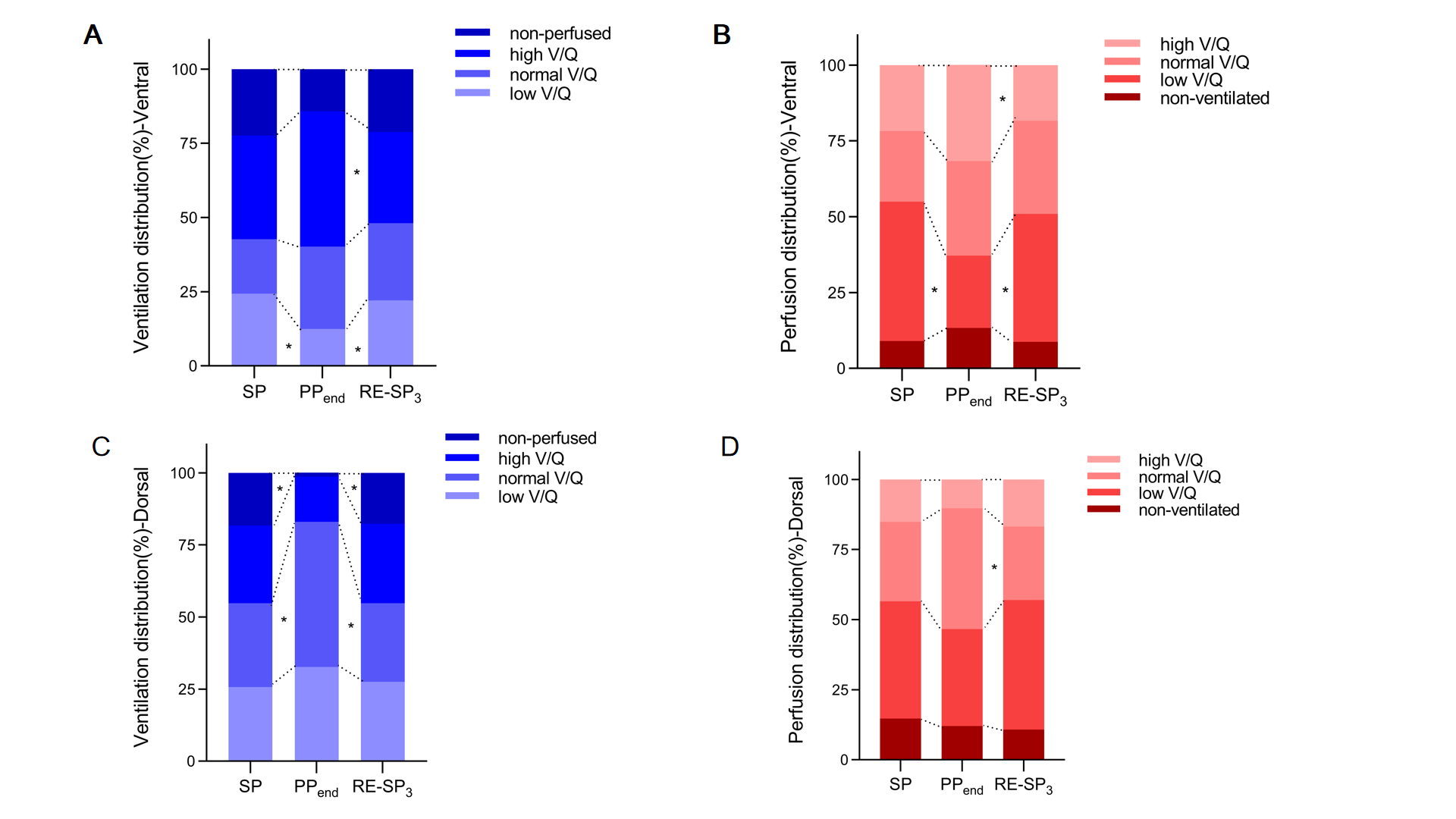
**

**Figure S3. Regional distribution of ventilation（A, C; blue bars）and perfusion(B, D; red bars) in the ventral(A, B) and dorsal (C, D) areas, across different ranges of V/Q before PP initiation (SP), at the end of PP(PP_end_), and 3 hours after returning to supine position (RE-SP_3_). *p < 0.05.**

**
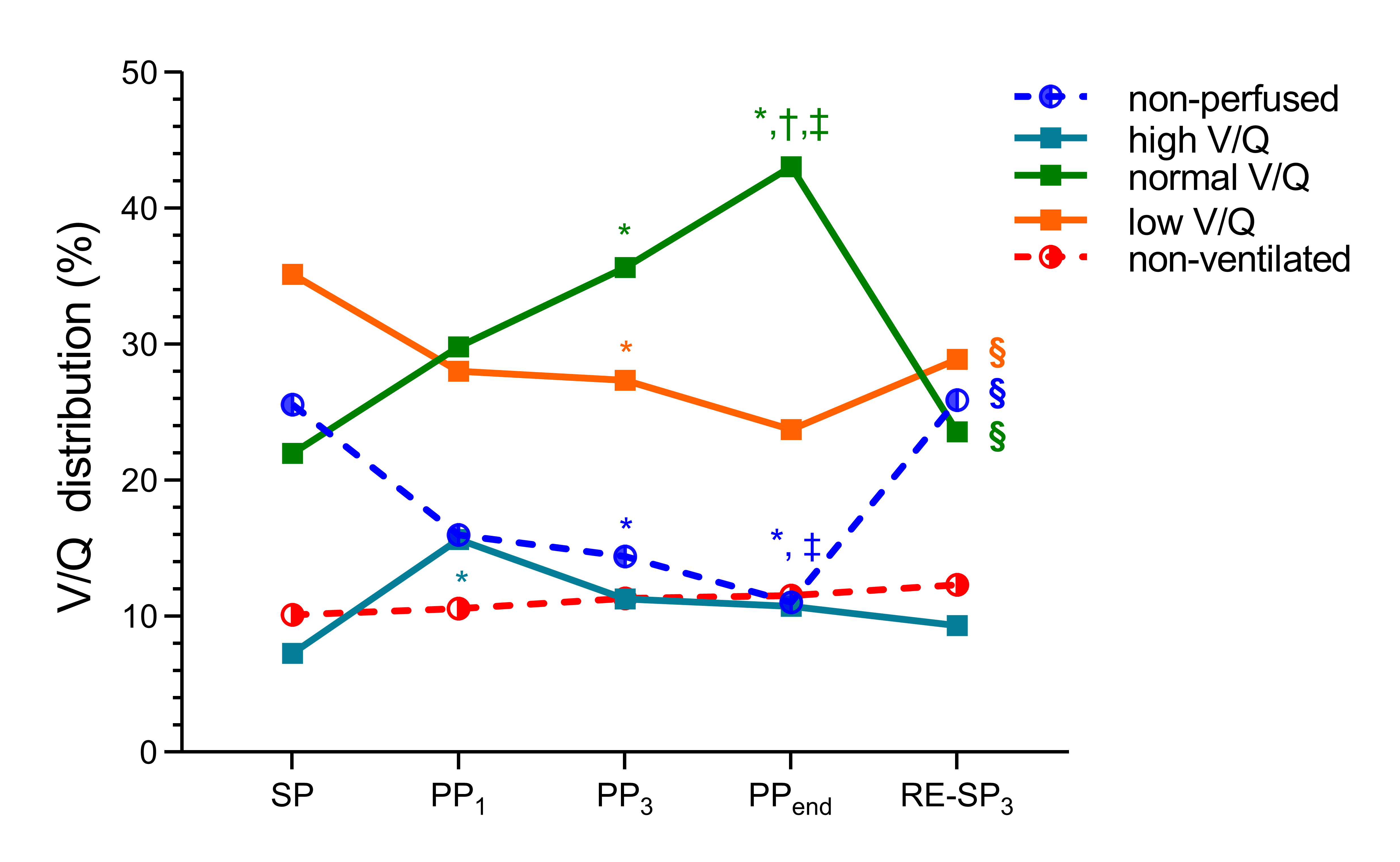
**

**Figure S4. Evolution of different V/Q distribution (%) before PP initiation (SP), 1 hour after PP (PP_1_), 3 hours after PP initiation (PP_3_), at the end of PP(PP_end_), and 3 hours after returning to supine position (RE-SP_3_). V/Q ratio was corrected by adiusting the V/Q-Rel values with the EIT-based novel calibration factor. * vs. SP,****p < 0.05, † vs. PP_1_, p < 0.05, ‡ vs. RE-SP_3_, p < 0.05.**

**Table S1. Fractions of different** **V/Q-Rel distributions along PP in the ventral and dorsal**

| **Variables** | **SP** | **PP_1_** | **PP_3_** | **PP_16_** | **RE-SP _3_** | **Trend*-P* value** |
| --- | --- | --- | --- | --- | --- | --- |
| **Pixels with-Ventral** |  |  |  |  |  |  |
| **Non-ventilated (V/Q≤0.1), %** | 3.16±4.10 | 5.53±5.12^*^ | 5.39±4.35^*^ | 4.42±2.25^*^ | 4.31±3.79 | **0.307** |
| **Low V/Q (0.1<V/Q<0.8), %** | 4.81±4.96 | 8.45±4.16 | 7.89±4.87 | 7.36±4.19 | 13.02±5.76^*^ | **<0.001** |
| **Normal V/Q (0.8≤V/Q≤1.25), %** | 8.77±5.52 | 13.04±7.23 | 13.00±8.46 | 10.99±7.26 | 11.58±5.73 | **0.196** |
| **High V/Q (1.25<V/Q<10), %** | 9.73±7.08 | 12.55±7.47 | 11.14±8.04 | 11.42±9.53 | 9.00±6.34 | **0.502** |
| **Non-perfused (V/Q≥10), %** | 12.40±7.04 | 5.02±3.06^*^ | 4.83±3.15^*^ | 4.82±3.31^*^ | 9.34±6.40 | **<0.001** |
| **Pixels with-Dorsal** |  |  |  |  |  |  |
| **Non-ventilated (V/Q≤0.1), %** | 6.83±6.36 | 5.86±3.72 | 5.56±6.70 | 4.96±5.15 | 9.22±6.88 | **0.219** |
| **Low V/Q (0.1<V/Q<0.8), %** | 20.27±8.52 | 10.36±6.40^*^ | 10.50±6.40^*^ | 9.19±7.25^*^ | 10.71±5.81^*^ | **<0.001** |
| **Normal V/Q (0.8≤V/Q≤1.25), %** | 9.73±6.99 | 12.25±7.78 | 14.91±7.53 | 28.58±13.65^*,^^†,‡^ | 8.83±4.95 | **<0.001** |
| **High V/Q (1.25<V/Q<10), %** | 5.18±7.05 | 20.39±15.62 | 19.26±14.21 | 9.52±11.38 | 11.80±13.49^*^ | **0.050** |
| **Non-perfused (V/Q≥10), %** | 19.12±12.67 | 6.57±8.10 | 7.44±7.55 | 8.74±7.88 | 12.18±12.06 | **0.219** |

SP: in the supine position; PP_1_: 1 hour after PP initiation; PP_3_: 3h after PP initiation; PP_end_: at the end of PP; RE-SP_3_ :3h after supine position.

^*^ vs. SP, *p* < 0.05, ^†^ vs. PP_1_, *p* < 0.05, ^‡^ vs. RE-SP_3_, *p* < 0.05.

**Table S2. Fractions of wasted ventilation and perfusion along PP in the ventral and dorsal**

| **Variables** | **SP** | **PP_1_** | **PP_3_** | **PP_16_** | **RE-SP _3_** | **Trend*-P* value** |
| --- | --- | --- | --- | --- | --- | --- |
| **Wasted ventilation, %-global** | 27.83±12.16 | 17.50±9.41 | 19.56±112.95 | 17.78±7.72^*,‡^ | 28.17±15.39 | 0.008 |
| **Wasted ventilation, %-ventral** | 17.44±7.28 | 13.28±8.71 | 15.44±12.62 | 14.89±8.25 | 16.56±10.76 | 0.666 |
| **Wasted ventilation, %-dorsal** | 10.39±8.74 | 4.22±4.78 | 4.11±4.90^*,‡^ | 2.89±3.94^*,‡^ | 11.61±8.95 | 0.001 |
| **Wasted perfusion, %-global** | 36.83±11.49 | 26.72±7.27^*^ | 23.67±7.35^*,‡^ | 18.17±7.20^*,†,‡^ | 34.50±9.70 | <0.0001 |
| **Wasted perfusion, %-ventral** | 14.44±6.03 | 12.11±7.40 | 11.56±5.58 | 9.22±6..52^*^ | 13.5±10.47 | 0.231 |
| **Wasted perfusion, %-dorsal** | 22.39±8.87 | 14.61±8.18^*,‡^ | 12.11±8.96^*,‡^ | 8.94±6.23^*,†,‡^ | 21.00±9.18 | <0.0001 |

SP: in the supine position; PP_1_: 1 hour after PP initiation; PP_3_: 3h after PP initiation; PP_end_: at the end of PP; RE-SP_3_ :3h after supine position.

^*^ vs. SP, *p* < 0.05, ^†^ vs. PP_1_, *p* < 0.05, ^‡^ vs. RE-SP_3_, *p* < 0.05.

**Table S3. V/Q-Corr variables**

| **Variables** | **SP** | **PP_1_** | **PP_3_** | **PP_16_** | **RE-SP_3_** | **Trend*-P* value** |
| --- | --- | --- | --- | --- | --- | --- |
| **MV/CO-EIT** | 0.636±0.138 | 0.656±0.159 | 0.711±0.275 | 0.683±0.159 | 0.687±0.224 | 0.495 |
| **Pixels with** |  |  |  |  |  |  |
| **Non-ventilated (V/Q≤0.1), %** | 10.10±11.58 | 10.57±10.80 | 11.30±9.47 | 11.51±10.13 | 12.33±8.01 | 0.859 |
| **Low V/Q (0.1<V/Q<0.8), %** | 35.13±13.50 | 28.01±14.60 | 27.37±15.61^*^ | 23.72±11.25 | 28.88±14.70 | 0.002 |
| **Normal V/Q (0.8≤V/Q≤1.25), %** | 21.96±6.85 | 29.83±12.13 | 35.64±13.51^*^ | 43.05±9.73^*,†,‡^ | 23.57±8.46 | <0.001 |
| **High V/Q (1.25<V/Q<10), %** | 7.26±7.05 | 15.64±9.21^*^ | 11.27±16.53 | 10.71±7.13 | 9.32±7.02 | 0.069 |
| **Non-perfused (V/Q≥10), %** | 25.55±11.54 | 15.96±10.53 | 14.41±9.53^*^ | 11.00±6.30^*,‡^ | 25.88±12.47 | <0.001 |
| **Wasted ventilation, %** | 31.00±14.34 | 17.72±17.13^*^ | 17.94±13.99^*^ | 14.67±8.44^*,‡^ | 31.22±18.50 | <0.001 |
| **Wasted perfusion, %** | 47.22±12.13 | 34.39±11.77^*^ | 32.00±11.69^*,‡^ | 29.22±9.90^*,‡^ | 44.67±11.40 | <0.0001 |
| **GI V/Q** | 0.882±0.064 | 0.786±0.149^*^ | 0.783±0.148 | 0.794±0.150^*^ | 0.857±0.084 | 0.009 |

SP: in the supine position; PP_1_: 1 hour after PP initiation; PP_3_: 3h after PP initiation; PP_end_: at the end of PP; RE-SP_3_ :3h after supine position.

^*^ vs. SP, *p* < 0.05, ^†^ vs. PP_1_, *p* < 0.05, ^‡^ vs. RE-SP_3_, *p* < 0.05.

**References:**

1. **Pavlovsky B, Pesenti A, Spinelli E, Scaramuzzo G, Marongiu I, Tagliabue P, Spadaro S, Grasselli G, Mercat A, Mauri T: Effects of PEEP on regional ventilation-perfusion mismatch in the acute respiratory distress syndrome. Crit Care 2022, 26(1):211.**
2. **Leali M, Marongiu I, Spinelli E, Chiavieri V, Perez J, Panigada M, Grasselli G, Mauri T: Absolute values of regional ventilation-perfusion mismatch in patients with ARDS monitored by electrical impedance tomography and the role of dead space and shunt compensation. Crit Care 2024, 28(1):241.**
